# New analogues of 13-hydroxyocatdecadienoic acid and 12-hydroxyeicosatetraenoic acid block human blood platelet aggregation and cyclooxygenase-1 activity

**DOI:** 10.1186/1752-153X-6-152

**Published:** 2012-12-10

**Authors:** Taghreed Hirz, Ali Khalaf, Nehme El-Hachem, May F Mrad, Hassan Abdallah, Christophe Créminon, René Grée, Raghida Abou Merhi, Aïda Habib, Ali Hachem, Eva Hamade

**Affiliations:** 1 Department of Biochemistry and Molecular Genetics, Faculty of Medicine, AUB, Beirut, POBox 11–236, Lebanon; 2 Département de Chimie et de Biochimie, Laboratoire de Chimie Médicinale et des Produits Naturels & PRASE, EDST Lebanese University, Hadath, Lebanon; 3 Institut des Sciences Chimiques de Rennes, Université de Rennes 1, CNRS UMR 6226, Avenue du Général Leclerc, 35042, Rennes Cedex, France; 4 iBiTec-S, Service de pharmacologie et d’immuno analyse, CEA Saclay - Bât. 532, 91191, Gif-Sur-Yvette cedex, France; 5 Génomique et Santé, Lebanese University, Hadath, Lebanon

**Keywords:** Cyclooxygenase-1, Anti-thrombotic, Inhibitors, Polyunsaturated fatty acid, Thrombosis

## Abstract

**Background:**

Thromboxane A_2_ is derived from arachidonic acid through the action of cyclooxygenases and thromboxane synthase. It is mainly formed in blood platelets upon activation and plays an important role in aggregation. Aspirin is effective in reducing the incidence of complications following acute coronary syndrome and stroke. The anti-thrombotic effect of aspirin is obtained through the irreversible inhibition of cyclooxygenases. Analogues of 12-hydroxyeicosatetraenoic acid and 13-hydroxyocatdecadienoic acid were shown previously to modulate platelet activation and to block thromboxane receptors.

**Results and discussion:**

We synthesized 10 compounds based on the structures of analogues of 12-hydroxyeicosatetraenoic acid and 13-hydroxyocatdecadienoic acid and evaluated their effect on platelet aggregation triggered by arachidonic acid. The structure activity relationship was evaluated. Five compounds showed a significant inhibition of platelet aggregation and highlighted the importance of the lipidic hydrophobic hydrocarbon chain and the phenol group. Their IC_50_ ranged from 7.5 ± 0.8 to 14.2 ± 5.7 μM (Mean ± S.E.M.). All five compounds decreased platelet aggregation and thromboxane synthesis in response to collagen whereas no modification of platelet aggregation in response to thromboxane receptor agonist, U46619, was observed. Using COS-7 cells overexpressing human cyclooxygenase-1, we showed that these compounds are specific inhibitors of cyclooxygenase-1 with IC_50_ ranging from 1.3 to 12 μM. Docking observation of human recombinant cyclooxygenase-1 supported a role of the phenol group in the fitting of cyclooxygenase-1, most likely related to hydrogen bonding with the Tyr 355 of cyclooxygenase-1.

**Conclusions:**

In conclusion, the compounds we synthesized at first based on the structures of analogues of 12 lipoxygenase metabolites showed a role of the phenol group in the anti-platelet and anti-cyclooxygenase-1 activities. These compounds mediate their effects via blockade of cyclooxygenase-1.

## Background

**Figure 1:**
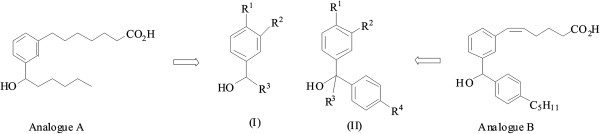
Design of the target molecules.

12-hydoxy-5Z,8Z,10E,14Z- eicosatetraenoic acid (12-HETE) and 13-hydroxy-9Z,11E octadecadienoic acid (13-HODE) are biologically active compounds derived from the metabolism of arachidonic acid and linoleic acid through the action of 12- and 15-lipoxygenase, respectively. These metabolites were shown to have anti-platelet effects mainly through inhibition of collagen and thromboxane-dependent aggregation for 12-HETE [1,2], 13-hydroperoxy-9Z,11E- octadecadienoic acid (13-HPODE) [3] and 13-HODE [4]. This later metabolite was also shown to decrease the adherence of platelets to endothelial cells [5]. The mechanisms by which these anti-platelet molecules affect platelet activation and aggregation involved inhibition of thromboxane (TX) synthesis or blocking of its receptors, ADP, thrombin and collagen receptors as well as blocking of glycoprotein –dependent binding of extracellular matrix and fibrinogen [6,7]. TXA_2_ is formed in blood platelets from arachidonic acid through the action of cyclooxygenase-1 (COX-1) and TX synthase. It acts on G-protein coupled receptors triggering platelet activation and aggregation [8]. COXs are membrane proteins that form homodimers [9]. X-Ray structure revealed a membrane protein with an active site in the interior globular part of the protein and a long non-polar channel as binding site for arachidonic acid and non steroidal anti-inflammatory drugs (NSAIDs) [10,11]. Arachidonic acid and acidic NSAIDS were shown to interact with Arg 120 and Tyr 355 of the active site of COX-1, where hydrogen bonds are involved in the interaction with the hydroxyl of Tyr 355 and carboxylate interaction were described with the amino residue of Arg 120 [12,13]. We have previously reported that stabilized analogues of 13- HODE (analogue A) and 12- HETE (analogue B) have inhibitory effects on TX –dependent platelet aggregation [14]. Starting from these analogues, we synthesized compounds of types I and II (Figure 1) and tested their effects on aggregation of human blood platelets in response to arachidonic acid, collagen and TX receptor agonist, and on COX-1 activity in cells overexpressing the recombinant human COX-1. We finally carried out modeling analysis of some compounds with COX-1.

## Results and discussion

### Chemistry

**Scheme 1:**
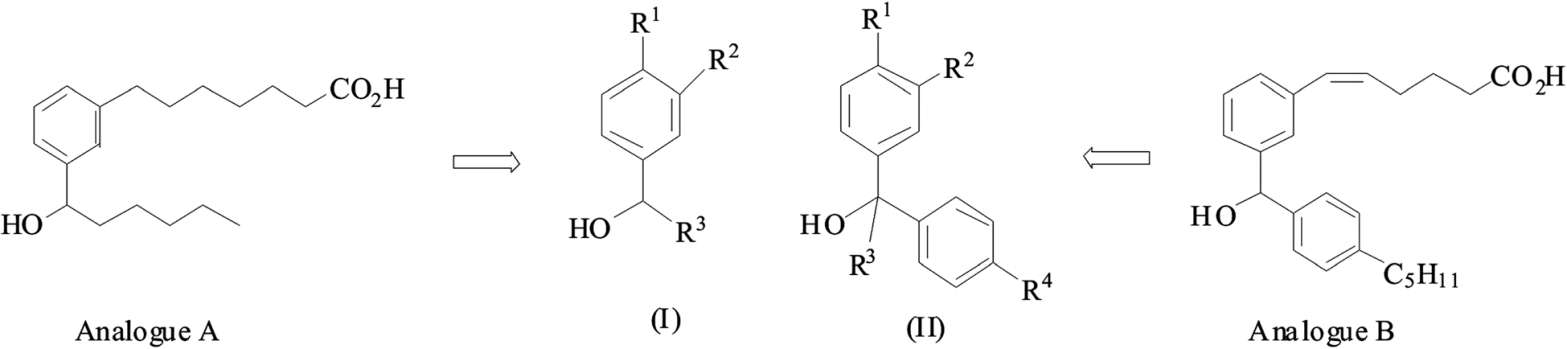
Preparation of compounds 1 and 2 from isophtalaldehyde.

Compounds **1** and **2** were obtained by removing the lipophilic chain with the carboxylic acid directly linked to the aromatic group (compound **2**) or replacing it by an oxime (compound **1**). We prepared these compounds from hydroxy-hexyl-benzaldehyde [14] starting from the commercial isophtalaldehyde (Scheme 1). The extension toward phenolic products is based on several literature data which show that polyphenols (natural and synthetic) are endowed with important anti-platelet aggregation activity [15-17]. Therefore we prepared new derivatives with OH and OMe groups on the aromatic core. These compounds were obtained by addition of the appropriate Grignard reagent to the suitable carbonyl compound.

### Biological activities

#### Effect on platelet aggregation

**Figure 2:**
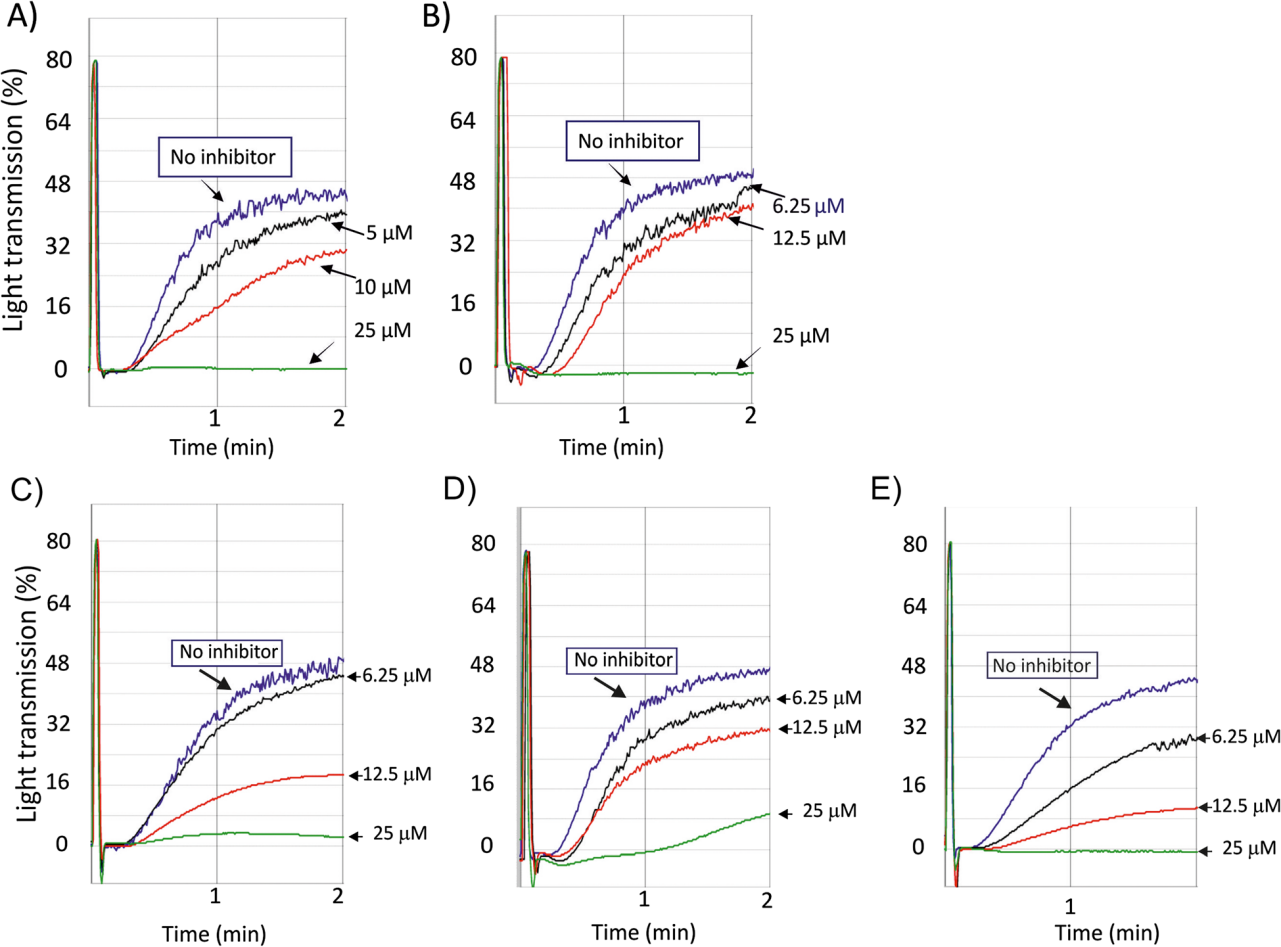
**Aggregation curves of inhibitory compounds.** Platelet aggregation was performed on human washed platelets. Platelets (0.4 × 10^9^ plt/ml) were treated with increasing concentrations of each inhibitor for 1 min prior to the addition of 25 μM of arachidonic acid for 2 min. Results represent aggregation curves of compound **3** (**A**), compound **4** (**B**), compound **7** (**C**), compound **8** (**D**), and compound **9** (**E**). Aggregation curves are representative of at least 4 experiments with similar results.

**Figure 3:**
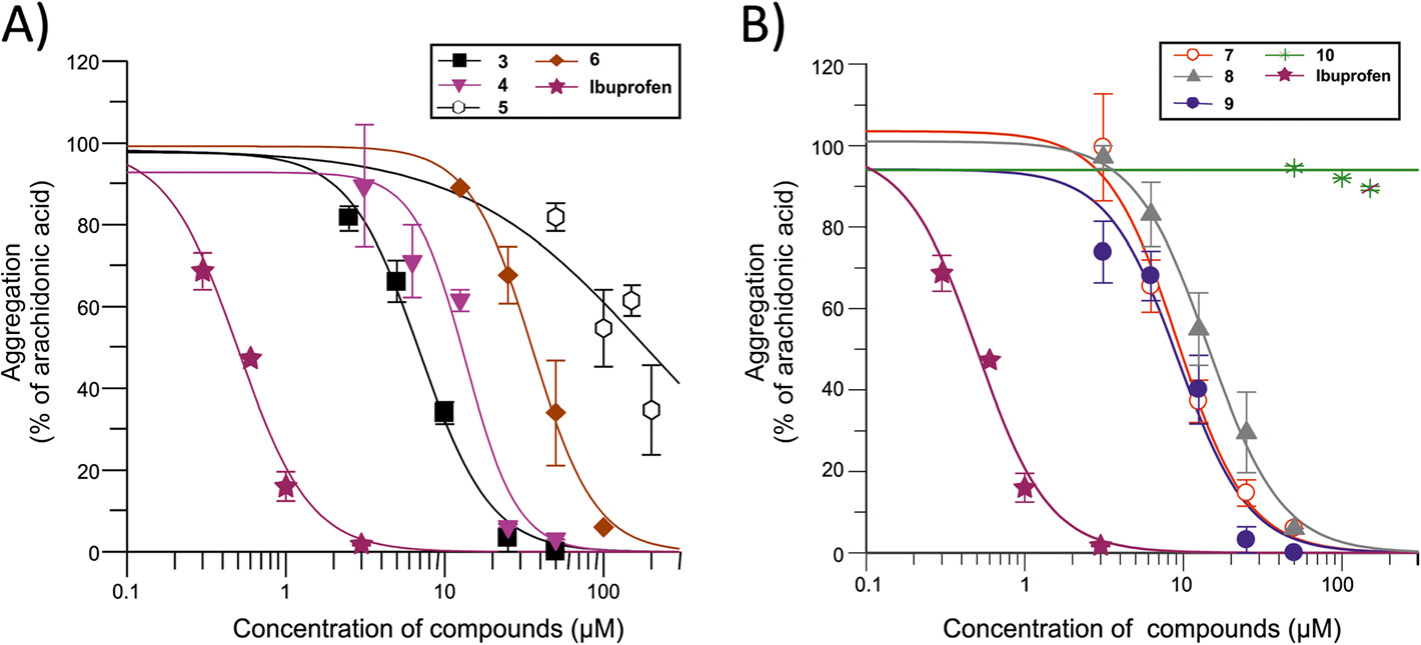
**IC**_**50 **_**fitting curves for inhibitory compounds and ibuprofen on platelet aggregation.** Slopes of aggregation curves were analyzed by Grafit 7 software and inhibition curves were obtained. (**A**) represents compounds of type I and (**B**) compounds of type II. Ibuprofen inhibitory curve is represented on each plot. Results correspond to mean ± S.E.M. for 3–5 experiments.

We evaluated the capacity of these compounds to block platelet aggregation triggered by arachidonic acid. We tested the role of the hydrophilic chain on the inhibitory activity profile of these compounds. Compounds **1** and **2** had a shorter hydrophilic carbonic chain compared to analogue A (Figure 1 and Scheme 1) and showed no inhibitory effect on arachidonic acid-dependent platelet aggregation (Table 1). We next evaluated the addition of substituents on the aromatic ring. Compound **3** with a hydroxyl at R^1^ and a methoxy at R^2^ was very potent with an IC_50_ of 7.5 ± 0.8 μM, mean ± S.E.M. (Table 1). Shortening the hydrophobic hydrocarbon chain at position R^3^ in compound **4** showed an IC_50_ of 14.2 ± 5.7 μM (n = 4) (Table 1) with a less clear dose–response effect on platelet aggregation by arachidonic acid. However, increasing the length of the hydrocarbon chain located at R^3^ to -C_9_H_19_ in compound **5** or replacing it with a cyclopentane group in compound **6** resulted in the loss of the inhibitory activity (Table 1). Figure 2A and 2B illustrate the aggregation curves of compounds **3** and **4**, respectively and figure 3A illustrates the IC_50_ curve fitting of compounds of type I. Ibuprofen was used as a reference for the inhibition of platelet aggregation (Figure 3A) and as expected, showed an inhibition of platelet aggregation with an IC_50_ of 0.5 ± 0.11 μM (n = 3). Our data suggest that the OH group at position R^1^ and a medium size length (C_5_H_11_) of the hydrophobic chain are involved in the inhibitory effect of the compounds of type I.

**Table 1:** Biological activity of compounds of type I and type II

**Structure**	**Compound**	**Type**	**R**^**1**^	**R**^**2**^	**R**^**3**^	**R**^**4**^	**Plt aggregation IC**_**50**_ **± S.E.M (μM)**^**b**^
	1		H	C = NOH	*n*C_5_H_11_	H	No inhibition
	2		H	COOH	*n*C_5_H_11_	H	No inhibition
	3	I	OH	OCH_3_	*n*C_5_H_11_	H	7.5 ± 0.8
	4		OH	OCH_3_	*n*C_4_H_9_	H	14.2 ± 5.7
	5		OH	OCH_3_	*n*C_9_H_19_	H	97.6 ± 83.5
	6		OH	OCH_3_		H	41.2 ± 0.7
	7		OH	OCH_3_	Ph	H	8.5 ± 1.2
	8	II	OH	OCH_3_	a	H	13.4 ± 0.5
	9		OH	OCH_3_	Ph	CH_3_	11.3 ± 4.8
	10		OCH_3_	OCH_3_	Ph	H	No inhibition

a = 4-Fluorophenyl; ^b^n = 3–4 in all experiments.

We next tested compounds of type II (Table 1). Compounds **7** and **8** exhibit significant inhibitory activities (Figure 2C and 2D, respectively) with compound **8** showing a more linear dose–response inhibition of the platelet aggregation (Figure 2D). Introduction of a methyl group on the carbinol centre (R^3^ group) did not change the inhibitory effect since compound **9** exhibited also the same range of inhibition with a similar linear dose–response inhibition (Figure 2E). On the contrary, replacing the hydroxyl group by a methoxy group in compound **10**, completely abolished the inhibitory activity (Figure 3B). These results support a role of the OH group in position R^1^. The IC_50_ of type II compounds were also compared with ibuprofen (Table 1). We conclude from these results that for type I molecules the hydroxyl group at R^1^ position is critical for the inhibitory effect and that the length of the R^3^ hydrocarbon chain is appropriate between 4 and 5 carbons, as described for compounds **3** and **4**, respectively. Type II compounds **7** and **9** are the most appropriate inhibitory compounds of this group with a hydroxyl group at R^1^ and a methoxy group at R^2^ position.

#### Effect on human COX-1 overexpressed in COS-7 cells

**Figure 4:**
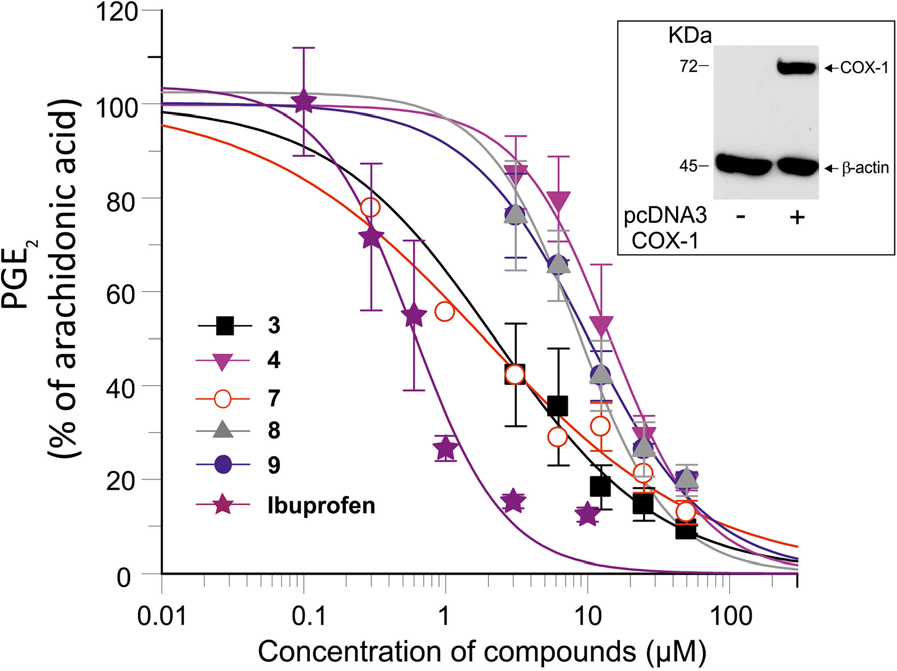
**Inhibition of COX-1 activity.** Human COX-1 was overexpressed in COS-7 cells. Cells were treated in the absence or presence of different concentrations of the compounds **3, 4, 7, 8** and **9** or ibuprofen prior to the addition of 25 μM arachidonic acid as indicated in the method section. PGE_2_ was measured in the supernatant by enzyme immunoassay. Results were expressed as percentage of PGE_2_ in arachidonic acid treated samples which was attributed a value of 100. Fitting of curves was done using Grafit software. Results correspond to mean ± S.E.M. for 3 experiments. (Insert) Cells were transfected in suspension with pcDNA3-COX-1 plasmid using Fugene® 6 transfection reagent at a ratio of 3:1 (fugene: DNA). Insert represents Western blot analysis of COX-1 using selective monoclonal COX-1 in untransfected and pcDNA3-COX-1transfected cells.

**Figure 5:**
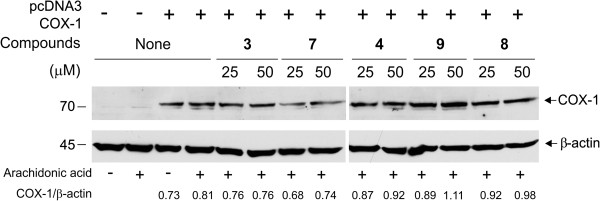
**Effect of compounds 3, 4, 7, 8 and 9 on COX-1 expression in COS-7 cells.** COS-7 transfected cells were incubated for 30 minutes with 25 or 50 μM of compounds **3, 4, 7, 8** and **9** prior to the addition of 25 μM of arachidonic acid (AA) for an additional 30 minutes, after which cells were lysed. Proteins of non-transfected and transfected cells were loaded on a 10% SDS-polyacrylamide gel and blotted for COX-1 and β-actin expression. Densitometry analysis was performed and ratio of COX-1/β-actin was calculated. Results are representative of 2 experiments with similar results.

We next verified that these compounds **3, 4, 7, 8** and **9** which block platelet aggregation affect directly COX-1. We used COS-7 cells overexpressing human COX-1. Figure 4 (insert) shows COX-1 protein overexpressed in COS-7 cells in comparison to non-transfected cells. In these cells which express only COX-1, arachidonic acid is metabolized into prostaglandin (PG) H_2_ which will consequently breakdown into PGE_2_. Measurement of PGE_2_ under these conditions will reflect COX-1 activity [18,19]. The IC_50_ values for the inhibition of COX-1 by the five different compounds are summarized in Table 2 and the fitting curves are presented in figure 4 along with the result of ibuprofen (IC_50_ was 0.5 ± 0.07 μM,, n = 3). All 5 compounds showed a strong and significant inhibition of the synthesis of PGE_2_ in COS-7 overexpressing human COX-1. No toxicity was observed with 25 or 50 μM of these compounds using WST-1 viability assay. Compounds **3, 4, 7, 8** and **9** showed an average of 120–130% of viability when compared to untreated cells, supporting the absence of toxicity of these compounds in the cell assay for COX-1. Moreover, COX-1 expression in COS-7 cells was not modified after treatment with the different molecules compared to untreated cells and to arachidonic acid treated cells, as shown by western blot and densitometric analysis (Figure 5).

**Table 2:** Effect on cyclooxygenase-1 activity

**Compound**	**COX-1 activity**
	**IC**_**50**_ **± S.E.M (μM)**^**a**^
**3**	2.2 ± 0.7
**4**	12.1 ± 2.3
**7**	1.3 ± 0.5
**8**	6.5 ± 0.2
**9**	8.1 ± 1.7

^a^ n = 3-4 in all experiments.

#### Effect on collagen- and thromboxane receptor - dependent platelet aggregation

**Figure 6:**
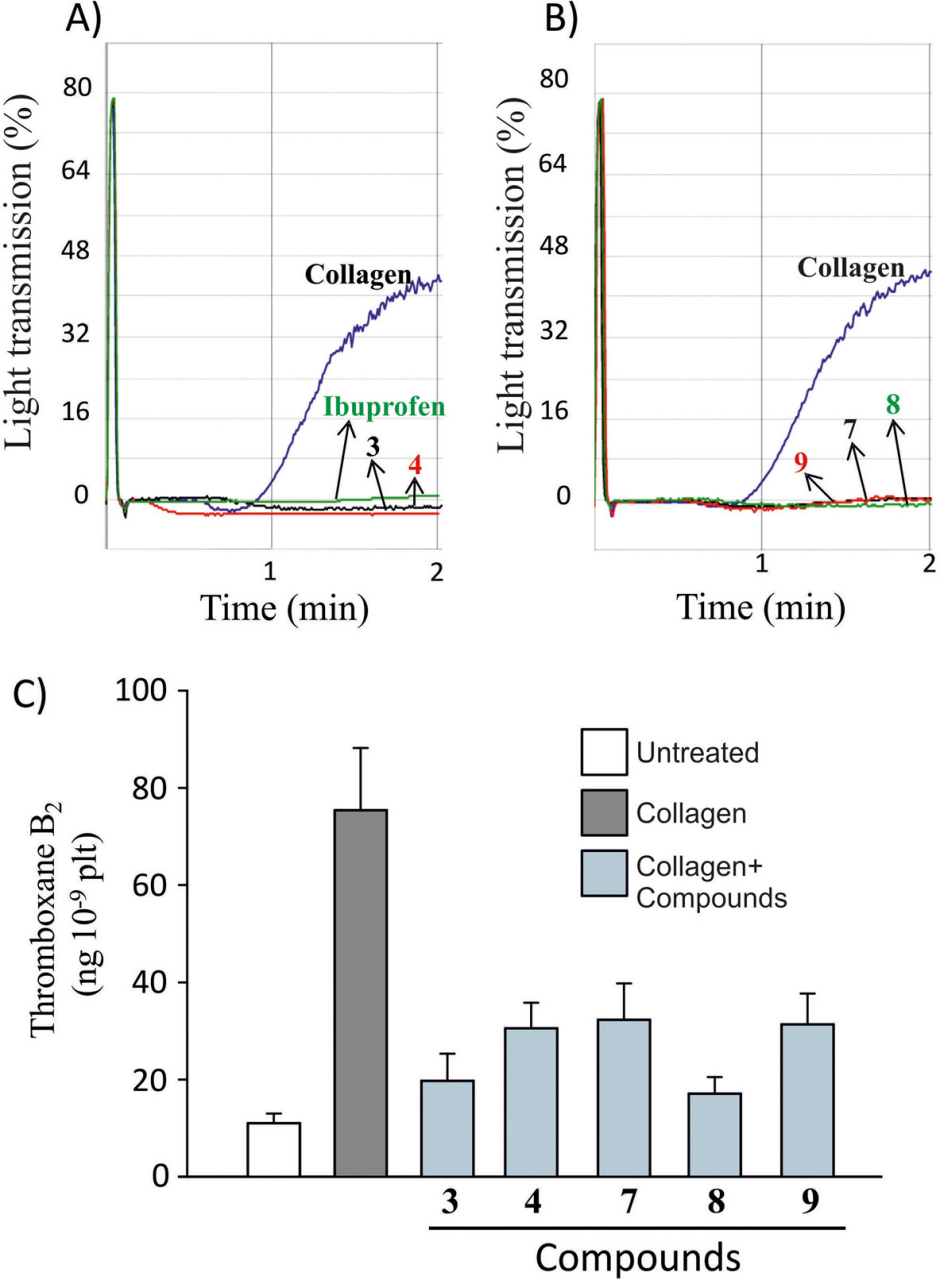
**Effect of inhibitory compounds on platelet aggregation and TXB**_**2 **_**level formation in response to collagen.** Platelets (0.4x 10^9^ plt/ml) were pretreated with 25 μM of ibuprofen or 50 μM of the inhibitory compounds **3, 4, 7, 8** and **9** then triggered with 0.5 μg/ml collagen. Results represent the aggregation curves of collagen with inhibitory compounds of type I and ibuprofen (**A**) and compounds of type II (**B**). TXB_2_ levels were measured in the supernatant of platelets (**C**). Results represent 3 experiments. Platelet aggregation by collagen alone versus collagen + inhibitory compounds treated-platelets showed a statistically significant difference (P < 0.02 One Way ANOVA- Bonferroni test).

**Figure 7:**
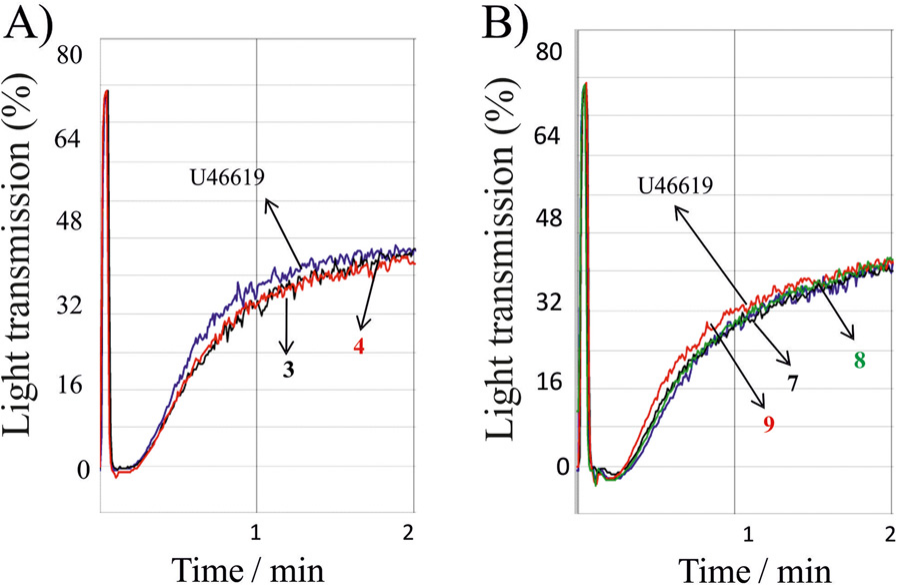
**Effect of inhibitory compounds on platelet aggregation in response to TX receptor agonist, U46619.** Platelets (0.4x 10^9^ plt/ml) were pretreated with 50 μM of inhibitory compounds **3, 4, 7, 8** and **9** then triggered with 1 μM U46619, a thromboxane receptor agonist. Aggregation curves of compounds of type I (**A**) and type II (**B**) are representative of 2 experiments with similar results.

We next tested the effect of the inhibitory compounds in the presence of collagen, a more physiological platelet agonist than arachidonic acid. Low concentration of collagen is described to bind collagen receptors linked to phospholipase A_2_ activation and arachidonic acid release [20]. In our conditions, platelet aggregation induced by 0.5 μg/ml, a low collagen concentration, was strongly inhibited by 25 μM ibuprofen (Figure 6A). Compounds **3** and **4** of type I series (Figure 6A) and compounds **7**, **8** and **9** of type II series (Figure 6B) blocked platelet aggregation induced by collagen. The effect of these compounds was also evaluated on collagen-dependent TX synthesis. TX concentration was increased 6–7 fold by collagen treatment as compared to untreated cells. The tested compounds strongly blocked collagen-dependent TX synthesis (Figure 6C). None of the compounds **3, 4, 7, 8** and **9** were able to act on the thromboxane receptor- dependent platelet aggregation in response to 1 μM of U46619, an agonist of the thromboxane receptor (Figure 7). Overall, these results, along with the effect on arachidonic acid- dependent platelet aggregation and on human recombinant COX-1, support that these new molecules affect platelet aggregation via inhibition of TX formation.

### Modeling analysis

**Figure 8:**
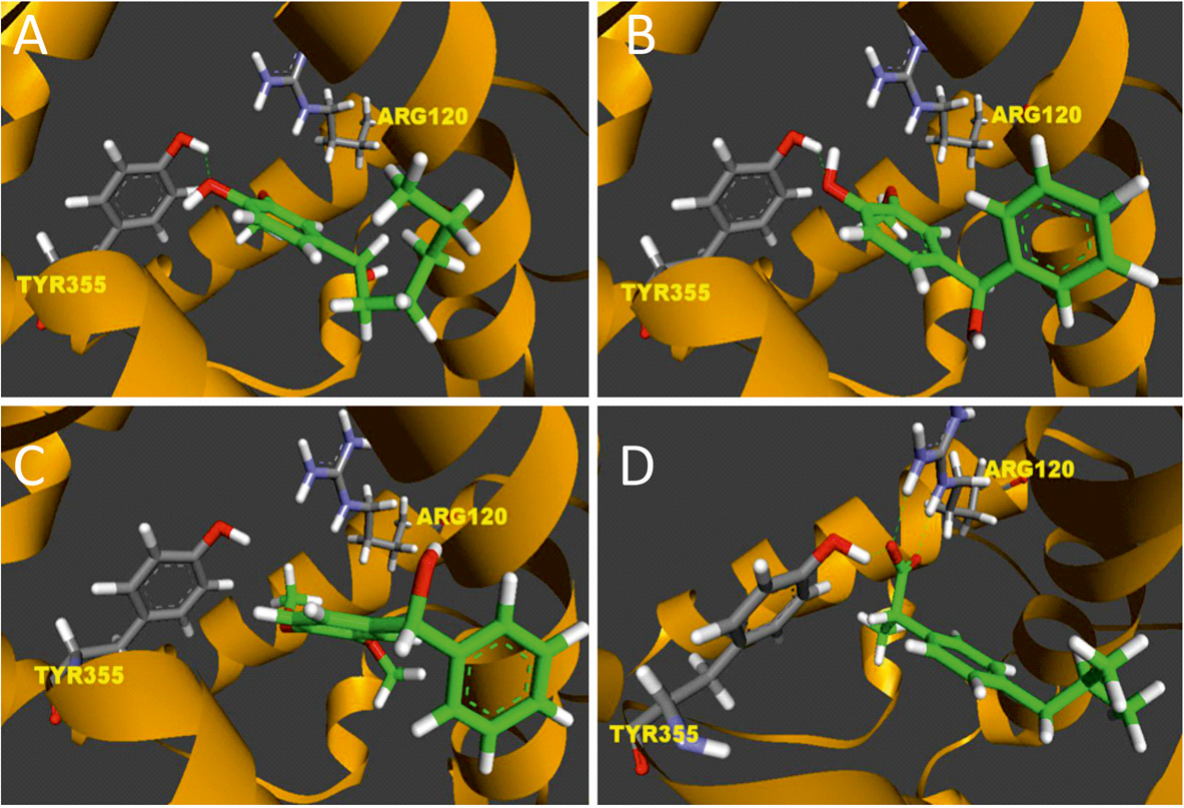
**Docking compounds into ovine COX-1.** The protein was rendered in orange ribbons. Compounds and relevant amino acids (Tyr 355 and Arg 120) in sticks (carbon atoms of compounds were colored in green). H-bonds are indicated as green dashed lines. Results for compounds **3** (**A**), **7** (**B**), **10** (**C**), and ibuprofen (**D**) are illustrated. The distances between the hydrogen donor and acceptor were estimated and were 2.30 angstroms between TYR355 and (OH) of compound **2**, 2.24 angstroms for TYR355 and (−OH) of compound **7** and 2.36 angstroms for TYR355 - ibuprofen.

We finally carried out modeling analysis of some of the inhibitors with ovine COX-1 to examine how these compounds dock the active site of the enzyme and to determine the amino acids of the active site of the enzyme involved in the interaction with the compounds. ibuprofen docked into COX-1and, in agreement with literature, the carboxyl group of ibuprofen showed three hydrogen bonds, two with Arg 120 (guanidine –NH_2_ group) and one with Tyr 355 (p-OH group) (Figure 8D). A root square mean deviation value of 1.2 Å was calculated between the best pose and the experimental coordinates of ibuprofen. Compounds **3** and **7** were docked nearby Tyr 355 and Arg 120. Both compounds exhibited hydrogen bond between phenol and Tyr 355 (Figure 8A and B). The binding scores of compounds **3** and **7** were −6.8 kcal/mol and −7.7 kcal/mol (−6.5 kcal/mol, and −7.6 kcal/mol for the S-enantiomers), respectively. These scores were comparable to ibuprofen (−8 kcal/mol). On the other hand, compound **10** which does not affect platelet aggregation (Figure 4B), did not interact with Tyr 355 (Figure 8C) although its binding score was −7.2 kcal/mol (−6.8 kcal/mol for the (S) enatiomer). Also, it is important to mention that compound 33 showed many docking poses that were far from the original binding site of ibuprofen compared to the active chemicals. Moreover, we docked rofecoxib, a selective COX-2 inhibitor and observed that the average root-mean-square deviation between the best reported pose for rofecoxib and the native compound ibuprofen was 7 angstroms, although its docking score was −7.2 kcal/mol (data not shown). It is well known that scoring functions yield high rates of false positives especially when the docked structures are very similar. Future experiments with enriched libraries of inactive compounds may improve docking and scoring performance. We conclude that Autodock succeeded in docking and scoring correctly active compounds **3** and **7**. It is very probable that the hydroxyl group at R^1^ is important for the interaction and the structure activity relationship of these compounds. Blocking this –OH interfered with the biological activity of these inhibitors.

## Conclusion

We first designed series of new aromatic compounds based on the structure of analogues of polyunsaturated fatty acid metabolites, 12-HETE and 13-HODE, we reported earlier [14]. These new compounds have important structural differences with the initial molecules analogues A and B. Five compounds were shown to have inhibitory effects on arachidonic acid- and collagen- induced platelet aggregation. These compounds mediate these effects via blockade of COX and not as antagonist of the TX receptor. Our results support a role of the phenol group in the inhibitory effect of these compounds, shown also by the docking observation of the molecules in the human recombinant COX-1. The addition of OH at R^1^ conferred additional properties and fitting in COX-1. There is a direct interaction of the compounds with COX-1 as revealed by the structure-activity relationship data, which showed an important role for the OH in position R^1^, most likely related to hydrogen bonding with the Tyr 355 of COX-1. In conclusion, the compounds we synthesized at first based on the structures of analogues of 12-HETE and 13-HODE show a role of the phenol group in the anti-platelet and anit-COX-1 activities. These molecules, although structurally different from the initial analogues of 12- HETE and 13-HODE compounds, have anti-platelet effect and anti- COX activities and will help to design more potent analogues. Studies are undertaken in our group and will be reported in due course.

## Methods

### General

COS-7 cells were obtained from the American Type Culture Collection (ATCC, Massas, VA). Ibuprofen was from EMD-Calbiochem (San Diego, CA). Arachidonic acid, monoclonal antibody anti-bactin and BSA were from Sigma-Aldrich (St Louis, MO). Collagen was from Chrono-Log Corp (Havertown, PA). PGE_2_ and TXB_2_ reagents were from Cayman Chemicals (Ann Arbor, MI). WST-1 viability assay and FuGENE® 6 transfection reagent were from Roche Applied Science (Indianapolis, IN.). All drugs were dissolved in DMSO and final concentration did not exceed 0.2%. Vehicle used was 0.2% DMSO and was added in controls or arachidonic treated platelets and COS-7 cells. All other chemicals and electrophoresis reagents were of high pure grade and were obtained from Amresco (Solon, OH) and BioRad (Hercules, CA).

### General procedure for preparation of compounds 3, 4, 5, 6, 7, 8, 9, and 10

In a glass vial equipped with a magnetic stirring bar and flashed with nitrogen gas aldehyde/ketone (1 equiv.) was dissolved in dry THF. The mixture was cooled at −15°C before the drop wise addition of the Organomagnesium reagent (1.3 equiv.). The reaction mixture was stirred under nitrogen gas for 1 hour, while the temperature rising slowly to room temperature. The mixture was treated with a saturated NH_4_Cl solution, extracted by ethyl acetate, dried over MgSO_4_, and concentrated in Vacuo. The adducts were obtained after silica gel column chromatography in (45 - 70% yield) with high purity.

### Preparation of compound 1

In a glass vial equipped with a magnetic stirring bar, hydroxy-hexyl-benzaldehyde (0.39 g, 1.86 mmol), NH_2_OH, HCl (0.2 g, 1.5 equiv.) and pyridine (1.2 ml) were dissolved in ethanol (8 ml). The mixture was refluxed overnight. After cooling to room temperature, the mixture was acidified by diluted HCl solution, extracted by ethyl acetate, dried over MgSO_4_, and concentrated in Vacuo. After silica gel column chromatography, oxime 6 was obtained as white crystals (0.31 g, 75% yield); melting point: 80°C. ^1^H NMR (CDCl_3_, 300 MHz) δ, ppm: 0.78 (t, 3H, J = 6.6Hz); 1.12-1.35 (m, 6H); 1.54-1.70 (m, 2H); 2.82 (bs, 1H); 4.57 (dd, 1H, J = 6.2, 7.0 Hz, 7.17-7.34 (m, 3H); 7.44 (s, 1H), 8.01 (s, 1H); 9.15 (bs, 1H). ^13^C NMR (CDCl_3_, 75 MHz) δ, ppm: 13.96; 22.50; 25.37; 31.66; 38.85; 74.42; 124.55; 126.22; 127.66; 128.80; 132.12; 145.45; 150.36.

### Preparation of compound 2

AgNO_3_ (200 mg) was dissolved in water before the addition of NaOH and formation of precipitates. Few drops of NH_3_ were added and the precipitates were totally dissolved. This mixture was added to aldehyde **3** in DMSO (3 ml) and stirred for 30 minutes. The mixture was acidified by diluted HCl solution and filtrated. The filtrate was extracted by ethyl acetate, dried over MgSO_4_, and concentrated in Vacuo. After silica gel column flash chromatography acid 10 was obtained as white crystals (70 mg, 44% yield); melting point: 110°C. ^1^H NMR (acetone, 300 MHz) δ, ppm: 0.73 (t, 3H, J = 7.05 Hz);1.10-1.39 (m, 6H); 1.49-1.67 (m, 2H); 4.61 (dd, 1H, J = 5.7, 7.2 Hz); 7.29-7.34 (m, 1H); 7.474-7.51 (m, 1H); 7.77 (td, 1H, J = 1.5, 7.7Hz); 7.93 (t, 1H, J = 1.7 Hz). ^13^C NMR (acetone, 75 MHz) δ, ppm: 14.29; 23.28; 26.13; 32.51; 40.48; 73.76; 127.96; 128.86; 129.03; 131.28; 131.34; 147.93; 167.83.

### Spectral and characterization data of compounds 3, 5, 6, 7, 8, 9 and 10

Compound **3:**^1^H NMR (CDCl_3_, 300 MHz) δ, ppm: 0.79 (t, 3H, J = 6.1 Hz); 1.20-1.36 (m, 6H); 1.53-1.62 (m, 1H); 1.64-1.72 (m, 1H); 3.80 (s, 3H); 4.49 (t, 1H, J = 6.7 Hz); 5.65 (s, 1H); 6.67-6.81 (m, 3H). ^13^C NMR (d_6_ acetone, 75 MHz) δ, ppm: 14.0; 22.6; 25.6; 31.7; 39.0; 55.9; 74.7; 108.4; 114.1; 119.0; 137.0; 145.0;146.6. MS: Found m/z: 224.

Compound **5** : ^1^H NMR (CDCl_3_, 300 MHz) δ, ppm: 0.77 (t, 3H, J = 6.85Hz); 1.09-1.21 (m, 14H); 1.37-1.46 (m, 1H); 1.61-1.74 (m, 1H); 3.80 (s, 1H); 3.86 (dd, J = 5.70, 7.66 Hz); 5.68 (s, 1H); 6.58 (dd, 1H, J 1.8 Hz, 8.0 Hz); 6.76 (d, 1H, J = 1.8 Hz); 6.78 (d, 1H, J = 8.0Hz). ^13^C NMR (CDCl_3_, 75 MHz) δ, ppm: 14.1; 22.7; 26.2; 29.3; 29.5; 29.6;31.9; 38.9; 55.8; 78.2; 109.0; 113.9; 120.3; 135.2; 144.9; 146.7.

Compound **6**: ^1^H NMR (CDCl_3_, 300 MHz) δ, ppm: 1.45-2.01 (m, 9H); 3.83 (s, 3H); 4.25 (d, 1H, J = 11.38Hz), 5.51 (s, 1H); 6.73 (dd, 1H, J = 1.78, 8.06 Hz); 6.80 (d, 1H, J = 8.06 Hz); 6.83 (d, 1H, J = 1.78 Hz). ^13^C NMR (CDCl_3_, 75 MHz) δ, ppm: 25.4; 25.6; 29.5; 29.8; 30.9; 47.6; 55.9; 79.2; 108.8; 114.0; 119.5; 136.6; 145.0; 146.6. MS: Found m/z: 222.

Compound **7**: ^1^H NMR (CDCl_3_, 300 MHz) δ, ppm: 2.45 (s, 1H); 3.70 (s, 3H); 5.65 (s, 1H), 6.74-6.85 (m, 3H); 7.17-7.33 (m, 5H). ^13^C NMR (CDCl_3_, 75 MHz) δ, ppm: 55.9; 76.0; 109.3; 114.2; 119.7; 126.4; 127.4; 128.4; 136.1; 144.0; 145.1; 146.7. MS: Found m/z: 230.

Compound **8**: ^1^H NMR (CDCl_3_, 300 MHz) δ, ppm: 3.80 (s, 3H); 4.87 (d, 1H, J = 3.93 Hz); 5.77 (d, 1H, J = 3.78 Hz); 6.79 (d, 1H, J = 8.10 Hz); 6.84 (d, 1H, J = 8.10 Hz); 7.04-7.10 (m, 3H); 7.43-7.48 (m, 2H). ^13^C NMR (CDCl_3_, 75 MHz) δ, ppm: 56.2; 75.38; 110.9; 115.4 (d, 2C, J_C-F_ = 21.3 Hz); 115.5; 120.2; 129.1 (d, 2C; J_C-F_ = 8.0 Hz); 137.8; 142.8 (d, 1C, J_C-F_ =3.0 Hz); 146.6; 148.2; 162.6 (d, 1C, J_C-F_ = 242.6 Hz). MS: Found m/z: 248.

Compound **9** : ^1^H NMR (d_6_ acetone, 300 MHz) δ, ppm: 1.92 (s, 3H); 3.19 (s, 1H); 3.77 (s, 3H); 4.60 (s, 1H); 6.78 (d, 1H, J = 8.25 Hz); 6.91 (dd, 1H, J = 2.07, 8.25 Hz); 7.13 (d, 1H, J = 2.07 Hz); 7.15-7.21 (m, 1H); 7.26-7.31 (m, 2H); 7.48-7.52 (m, 2H). ^13^C NMR (d_6_ acetone, 75 MHz) δ, ppm: 31.5; 56.3; 75.9; 110.9; 115.0; 119.5; 126.7; 127.0; 128.6; 141.9; 146.0; 147.8; 150.7. MS: Found: m/z =244.

Compound **10** : ^1^H NMR (CDCl_3_, 300 MHz) δ, ppm: 3.76 (s, 3H); 3.78 (s, 3H); 5.71 (s, 1H); 6.74 (d, 1H, J = 8.19 Hz); 6.79-6.85 (m, 2H); 7.18-7.29 (m, 5H). ^13^C NMR (CDCl_3_, 75 MHz) δ, ppm: 55.8; 55.9; 75.9; 109.8; 110.9; 119.0; 126.4; 127.5; 128.5; 136.6; 143.9; 148.4; 149.0. MS: Found m/z: 244.

### Blood collection, platelet preparation and analysis

Venous blood was obtained from healthy volunteers who had not ingested any drugs for the last 14 days and after informed consent in accordance with the Institutional Review Board (IRB) of the American University of Beirut (Approval # BioCh.AH.03). Washed platelets were prepared as described previously [21,22]. Briefly, 20 ml of peripheral blood was withdrawn on ACD-C (1 volume for 9 volumes of blood) and centrifuged at 120 g for 15 min at room temperature (RT) to obtain platelet-rich plasma. Platelet-rich plasma was further centrifuged at 1,200 g for 15 min at RT, and the platelet pellet obtained was washed by Tyrode buffer solution containing 0.1 μM of PGE_1_ and further centrifuged at 1,200 g for 15 min at RT. The pellet was resuspended in Hanks buffer, pH 7.4 containing 1 mg/ml of bovine serum albumin. Aggregation of washed-platelets was determined using light transmittance aggregometry (Chrono-Log Corp., Havertown, PA). 400 μl of platelets (0.4 x 10^9^plt/ml) were preincubated for 1 min at 37^0^C in the absence or presence of inhibitors prior to the addition of 25 μM of arachidonic acid, which was controlled as optimal in our conditions. In some experiments, platelets were triggered with 1 μM U46619, a TX receptor agonist, or 0.5 μg/ml collagen, a concentration we checked in our conditions to induce COX-dependent platelet aggregation. TXB_2_ was measured in the supernatant by enzyme immunoassay [23].

### Overexpression of human recombinant COX-1 in COS-7 cells and COX-1 activity

COS-7 cells were grown using DMEM-medium containing 10% fetal bovine serum (FBS). Cells were transfected in suspension with pcDNA3-COX-1 plasmid using FuGENE® 6 transfection reagent at a ratio of 3:1 (FuGENE: DNA, v/w) and then cultured in DMEM media + 10% FBS in 12-well plates. For COX-1 activity, cells were incubated 48 hours post transfection in the absence or presence of different concentrations of compounds **3, 4, 7, 8** and **9** or ibuprofen in Hanks buffer, pH 7.4, containing 1 mg/ml BSA for 30 minutes prior to the addition of 25 μM arachidonic acid for 30 minutes. PGE_2_ was measured in the supernatants by enzyme immunoassay [23]. Cells were washed twice with PBS and lysed in lysis buffer. COX-1 protein was determined by western blot as previously described [24]. Briefly, SDS polyacrylamide electrophoresis was performed using 8% gels followed by protein transfer using a semi-dry transfer machine. Immunoblot analysis was performed using selective monoclonal antibody anti COX-1 (COX-111) (1/1000) [25] and a monoclonal antibody anti-β-actin (1/2000). The effect of 25 and 50 μM of compounds **3, 4, 7, 8** and **9** on cell viability of COS-7 cells overexpressing COX-1 was evaluated using WST-1 assay and showed an absence of toxicity of these compounds.

### Data analysis

Aggregation data were expressed after defining the slope for each aggregation curve, which better reflects the rate of the platelet reaction, using the Born’s method [26]. Curve fitting and calculation of the IC_50_ values were done using Grafit 7 software (Erithacus software, Staines, UK). Results of TXB_2_ and PGE_2_ measurement were expressed as the mean ± S.E.M. for at least 3 different experiments and statistical analysis was performed using Sigma Plot (Systat Software Inc., San Jose, CA). Autoradiograms obtained from western blot analyses were scanned using Epson 1680 pro scanner and densitometric analysis was performed using Scion Image (Scion Corporation, MD).

### Target selection and preparation

The 3D structure of the ovine COX-1 complexed with ibuprofen (PDBID: 1EQG) was selected for docking simulations [27]. Water and other heteratoms were removed from the structure. Chain A was retained including ibuprofen and heme group. Hydrogen atoms were added, atom typing and partial charges were assigned using AMBER forcefield [28]. The coordinates of the binding site were extracted using the co-crystallized ligand, Ibuprofen. Docking and scoring: low energy conformations of the chemical compounds were generated using Catalyst (Accelrys, Inc.). The (R)- enantiomers of compounds **3, 7,** and **10** were selected, docking simulations were carried out using Autodock 4.2 [29], each docking simulation was achieved with 10 docking runs with 150 individuals using the Lamarckian genetic algorithm implemented in Autodock and 250000 energy evaluations. The binding energies were estimated from a new free-energy scoring function based on the AMBER forcefield, an updated charge-based desolvation term and improved models of the unbound state. The best poses were analyzed and visualized with Disovery Studio visualizer (Accelrys, Inc.).

## Abbreviations

COX: Cyclooxygenase; TX: Thromboxane; PG: Prostaglandin; 12-HETE: 12-hydoxy-5Z,8Z,10E,14Z-eicosatetraenoic acid; 13-HODE: 13-hydroxy-9Z,11E-octadecadienoic acid; NSAIDs: Non-steroidal anti-inflammatory drugs; FBS: Fetal bovine serum; Plt: Platelet; AA: Arachidonic acid.

## Competing interests

The authors declare that they have no competing interests.

## Authors’ contributions

AK, HA, AHachem and RG designed the molecules and carried out the synthesis. TH, MFM, RAM, AHabib and EH designed and carried out the biological tests. NH performed docking analysis. CC assisted in setting up the prostaglandin and thromboxane assay and COX-1 detection. AHachem, A Habib, RG and EH conceived the study and participated in problem solving. All authors read and approved the final version of the manuscript.
